# Preservation of the integrity of facial nerve in vestibular schwannoma microsurgery: A consecutive study of 127 clinical cases focusing on nervus intermedius

**DOI:** 10.3389/fonc.2023.939983

**Published:** 2023-02-09

**Authors:** Yue Li, Hao Peng, Sen Zhang, Wenyong Long, Yimin Pan, Yang Li, Changwu Wu, Kai Xiao, Xiangyu Wang, Jun Su, Chaoying Qin, Qing Liu

**Affiliations:** ^1^ Department of Neurosurgery in Xiangya Hospital, Central South University, Changsha, Hunan, China; ^2^ Department of Neurosurgery, Hainan General Hospital, Haikou, Hainan, China; ^3^ Department of Neurosurgery, Hunan Children’s Hospital, Changsha, Hunan, China; ^4^ Institute of Skull Base Surgery & Neuro-oncology at Hunan Neurosurgery Institute of Central South University, Changsha, Hunan, China

**Keywords:** vestibular schwannoma, nervus intermedius, microsurgery, neurofunction preservation, surgical technique

## Abstract

**Background:**

Nervus intermedius (NI) injuries are not given enough attention by neurosurgeons during vestibular schwannoma (VS) surgery. Preservation of NI function is essential for the integrity and continuity of the facial nerve, although this can be challenging. We identified the risk factors for NI injury and proposed our experience for optimizing NI preservation based on our cases.

**Methods:**

We retrospectively analyzed clinical data from a consecutive series of 127 patients with VS who underwent microsurgery *via* the retrosigmoid approach from 2017 to 2021 at our institution. The baseline characteristics of the patients were collected from the medical records, and the incidence of NI dysfunction symptoms was obtained by outpatient and online video follow-up 6 months after surgery. The surgical procedures and techniques used were described in detail. The data were analyzed in relation to sex, age, tumor location (left or right), Koos grading scale, internal acoustic canal (IAC) invasion (TFIAC Classification), brainstem adhesion, tumor characteristics (cystic or solid), tumor necrosis, and preoperative House–Brackmann (HB) grading by univariate and multivariate analyses.

**Results:**

Gross tumor removal was achieved in 126 (99.21%) patients. Subtotal removal was performed on one patient (0.79%). Twenty-three of our cases exhibited facial nerve palsy preoperatively; 21 patients had HB grade II facial palsy, and two had HB grade III. Two months after surgery, 97 (76.38%) patients had normal function of the motor portion of the facial nerve; 25 (19.69%) patients had HB Grade II facial palsy, five had Grade III (3.94%), and zero (0%) had Grade IV. Postoperatively, 15 patients experienced newly gained dry eyes (11.81%), whereas 21 cases of lacrimal disturbances (16.54%), nine of taste disturbances (7.09%), seven of xerostomia (5.51%), five of nasal hypersecretions (3.94%), and seven of hypersalivation (5.51%) were identified in our cases. Univariate and multivariate analyses revealed that the Koos grading scale and tumor characteristics (solid or cystic) were correlated with NI injury (p <0.01).

**Conclusion:**

The data in this study demonstrate that although the motor function of the facial nerve is well preserved, NI disturbance is still common after VS surgery. Maintaining the integrity and continuity of the facial nerve is key to NI function. Performing bidirectional and subperineurium dissection based on even and adequate debulking is beneficial for NI preservation in VS surgery. Higher Koos grading and cystic characteristics of VS are associated with postoperative NI injuries. These two parameters can be used to guide the delineation of surgical strategy and predict the prognosis of NI function preservation.

## Introduction

Vestibular schwannomas (VSs) account for approximately 8% of all intracranial tumors and are the most common neoplasm of the cerebellopontine angle (CPA) in adults, with a lifetime prevalence exceeding one case among 500 individuals (1). Treatment strategies for VS can be divided into the observational wait-and-scan approach, irradiation, microsurgery, and a combination of these methods (1). Microsurgery remains the main treatment for most patients with VS. With the development of microsurgical dissection over the past century, the management of VS has moved beyond pursuing low morbidity and has resulted in almost no risk of death (2–4). Neurofunction and quality of life have been prioritized by neurosurgeons for clinical management.

Preservation of facial nerve (CN VII) functions is always one of the main challenges in the surgical management of VS. The majority of the current literature on the surgical outcome of VS resection focuses on the motor function of the facial nerve, since facial palsy leads to severe functional disability and psychological trauma (5, 6). However, intraoperative protection of the sensory/parasympathetic part of CN VII, which courses in the CPA cistern between the motor portion of the facial nerve and its various origins from the brainstem to the internal acoustic canal (IAC)–nervus intermedius (NI), has not gained sufficient attention. The NI has different connections with adjacent nerves in the CPA. The various orientations, tortuous trajectories, and flexible anastomoses of the NI make its texture different from that of the motor branches of the facial nerve. In contemporary reports, there are limited references to the postoperative function of the non-motor components of the facial nerve. Injury to the NI leads to dysfunction of the lacrimal gland, submandibular and sublingual salivary glands, and fibers of sensory perception in the skin of the external auditory meatus and nasopharynx. NI abnormalities are considered by neurosurgeons to be less severe than the motor dysfunction caused by damaged CN VII; nevertheless, disturbances of tear secretion and sensation of taste indeed plague patients who suffer from these symptoms. Notably, NI is found to have no perineurium and is generally covered with only a thin layer of arachnoidea (7), making it difficult to distinguish and protect NI intraoperatively (8, 9). Thus, we believe that the preservation of NI function is essential for obtaining the integrity and continuity of facial nerves.

The presence of NI injury symptoms following VS surgery inspired us to conduct this study. We obtained clinical data on the preoperative and postoperative functions of the NI from 127 patients with VS who underwent microsurgery *via* the retrosigmoid approach at our institution. Univariate and multivariate analyses were used to assess risk factors associated with postoperative NI function, such as sex, age, tumor location, Koos grading scale, IAC invasion (TFIAC Classification), brainstem adhesion, tumor characteristics (cystic or solid), tumor necrosis, and House–Brackmann (HB) grading. Based on the analyses, we summarized the surgical technique and our experience in optimizing the intraoperative protection of the NI. Our study could provide evidence for analyzing the risk of NI injury preoperatively and depicting the strategy for preserving the NI during VS resection.

## Materials and methods

### Study population

This retrospective study included a series of patients who achieved total or subtotal removal with pathological results of schwannoma after microsurgery *via* the retrosigmoid approach at Xiangya Hospital between January 2017 and April 2021. Outpatient follow-up was performed for these patients 6 months after their surgery, while online video follow-ups were performed if the patients had difficulty with outpatient follow-up in person.

### Clinical indicators

In addition to the standard epidemiological features of each patient, the following preoperative and postoperative symptoms were also documented: facial palsy, dry eyes, lacrimal disturbances, taste disturbances, xerostomia, nasal hypersecretion, and hypersalivation. Questions relating to the presence of symptoms and their characteristics were included for every symptom. A surgical approach was also used. Tumor-related data were also collected, and these parameters included sex, age, tumor location, Koos grading scale, IAC invasion (TFIAC Classification), brainstem adhesion, tumor characteristics (cystic or solid), tumor necrosis, and HB grading.

### Facial nerve motor function and tumor grade

Facial nerve motor function was assessed before and after surgery using the HB facial nerve outcome scale (10). Classification by size was based on the Koos grading system from grades I to IV as follows: grade I, a small intracanalicular tumor; grade II, a small tumor with protrusion into the CPA cistern; grade III, a tumor occupying the CPA cistern with no brainstem displacement; and grade IV, a large tumor with brainstem and cranial nerve displacement. IAC invasion was classified by TFIAC (tumor filling inner auditory canal) as follows: grade I, 0% < TFIAC ≤ 25%; grade II, 25% < TFIAC ≤ 50%; grade III, 50% < TFIAC ≤ 75%; grade IV, 75% < TFIAC ≤ 100% (11).

### Statistical analysis

Statistical analyses were performed using SPSS for Windows (IBM SPSS Statistics for Windows, Version 25.0, Armonk, NY: IBM Corp.). A descriptive analysis was conducted on the epidemiological and tumor-related data, as well as the postoperative symptoms referred to in the questionnaire and telephonic interview 2 months after surgery. The data were analyzed in relation to sex, age, tumor location (left or right), Koos grading scale, IAC invasion (TFIAC classification), brainstem adhesion, tumor characteristics (cystic or solid), tumor necrosis, and preoperative HB grading. To evaluate these data, either a univariate analysis or a t-test for multiple linear regression was used. Statistical significance was set at a probability value of <0.05.

## Results

### Patient characteristics

Detailed characteristics of the 127 patients are presented in **Table 1**. There were 82 females and 45 males (the female-to-male ratio was 1:0.55). The median age of the patients was 49 years (range, 13–73 years). The median follow-up period was 36 months (range, 6–118 months). The median tumor size was 43 mm (range, 12–98 mm). The lesions were all located in the CPA region and were diagnosed pathologically as schwannomas. In total, 41 cases were cystic and 86 cases were solid. A tumor hemorrhage occurred in 55 patients. All 127 patients (100%) had primary VSs. A total of 109 lesions (85.83%) exhibited significant adhesion to the brainstem. IAC invasion grade was determined by TFIAC classification (12). According to the grading system by Koo et al., there were two grade I, 17 grade II, 41 grade III, and 67 grade IV invasions in our series. In total, nine patients complained of NI abnormalities prior to surgery (**Table 1**).

**Table 1:** Baseline characteristics of 127 patients with VS.

Variable	No.of patients
Overall	127(100%)
Sex	
Male	45(35.43%)
Female	82(64.57%)
Age (years)	
≤50	71(55.91%)
>50	56(44.09%)
Confidence intervals	48.9291±1.08875
Tumor location	
Left	77(60.62%)
Right	50(39.37%)
Koos grading scale	
Grade I	2(1.57%)
Grade II	17(13.39%)
Grade III	41(32.28%)
Grade IV	67(52.76%)
Brainstem adhesion	
Yes	109(85.83%)
No	18(14.17%)
Solid or cystic tumor	
Solid	86(67.72%)
Cystic	41(32.28%)
Tumour necrosis	
Yes	55(43.31%)
No	72(56.69%)
TFIAC	
I	16(12.6%)
II	15(11.8%)
III	26(20.5%)
IV	70(55.1%)
Preoperative HB grading	
I	104(81.89%)
II	21(16.54%)
III	2(1.57%)
IV	0
Extent of resection	
GTR	126(99.21%)
STR	1(0.79%)

VS, Vestibular schwannoma.

HB, House-Brackmann.

GTR, Gross tumor resection.

STR, Subtotal tumor resection.

### Surgical management

**Figure 1:**
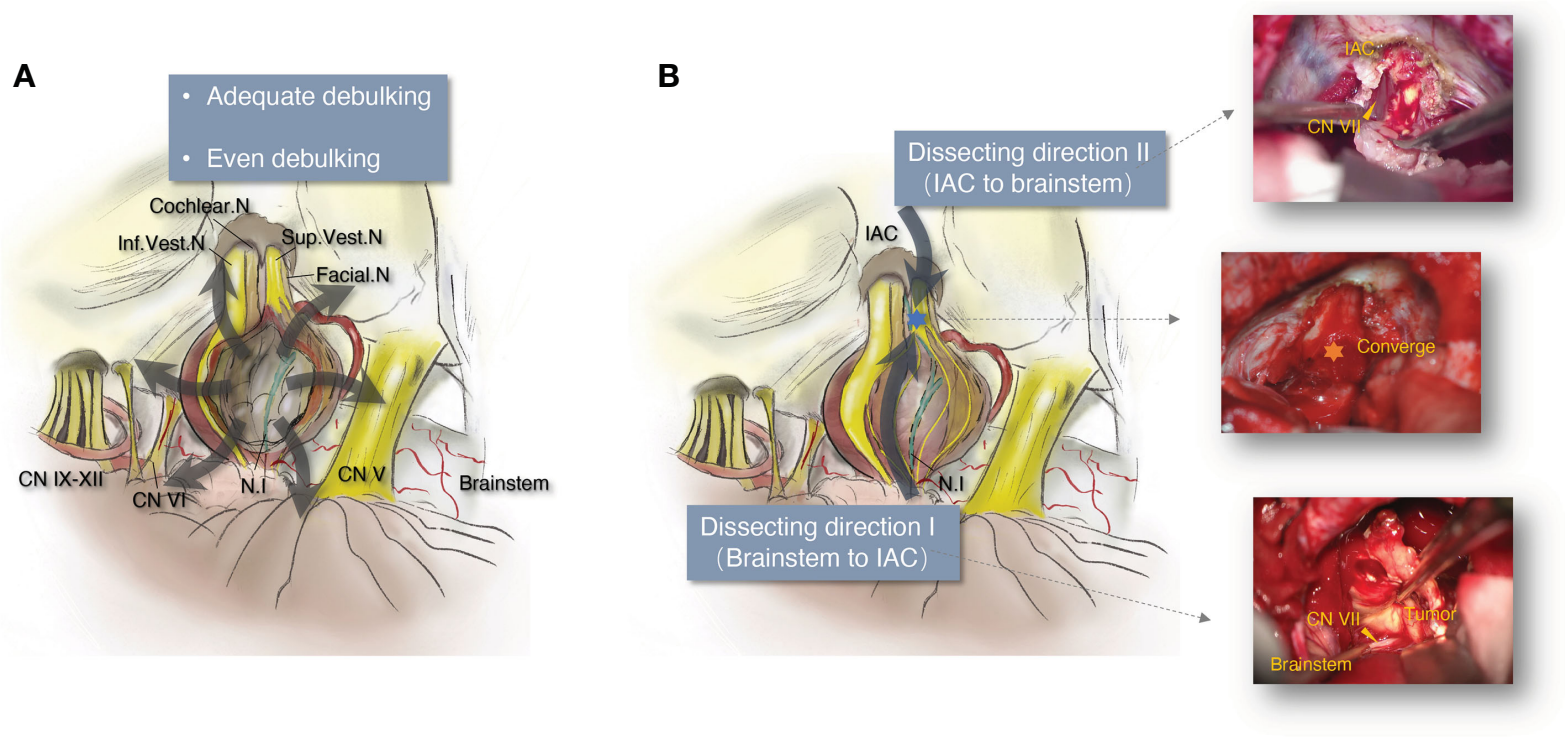
The schematic diagram illustrating the principle of vestibular schwannoma microsurgery for neurofunction preservation. **(A)** Schematic diagram of even and adequate debulking in vestibular schwannoma microsurgery. **(B)** Schematic diagram and intraoperative images of bidirectional dissection in vestibular schwannoma microsurgery. IAC, internal acoustic canal; Cochlear. N, cochlear nerve; Facial. N, facial nerve; Sup.Vest.N, superior vestibular nerve; Inf.Vest.N, inferior vestibular nerve; CN V, the 5th cranial nerve; CN VI, the 6th cranial nerve; CN IX–XII, the 9th to 12th cranial nerves.

**Figure 2:**
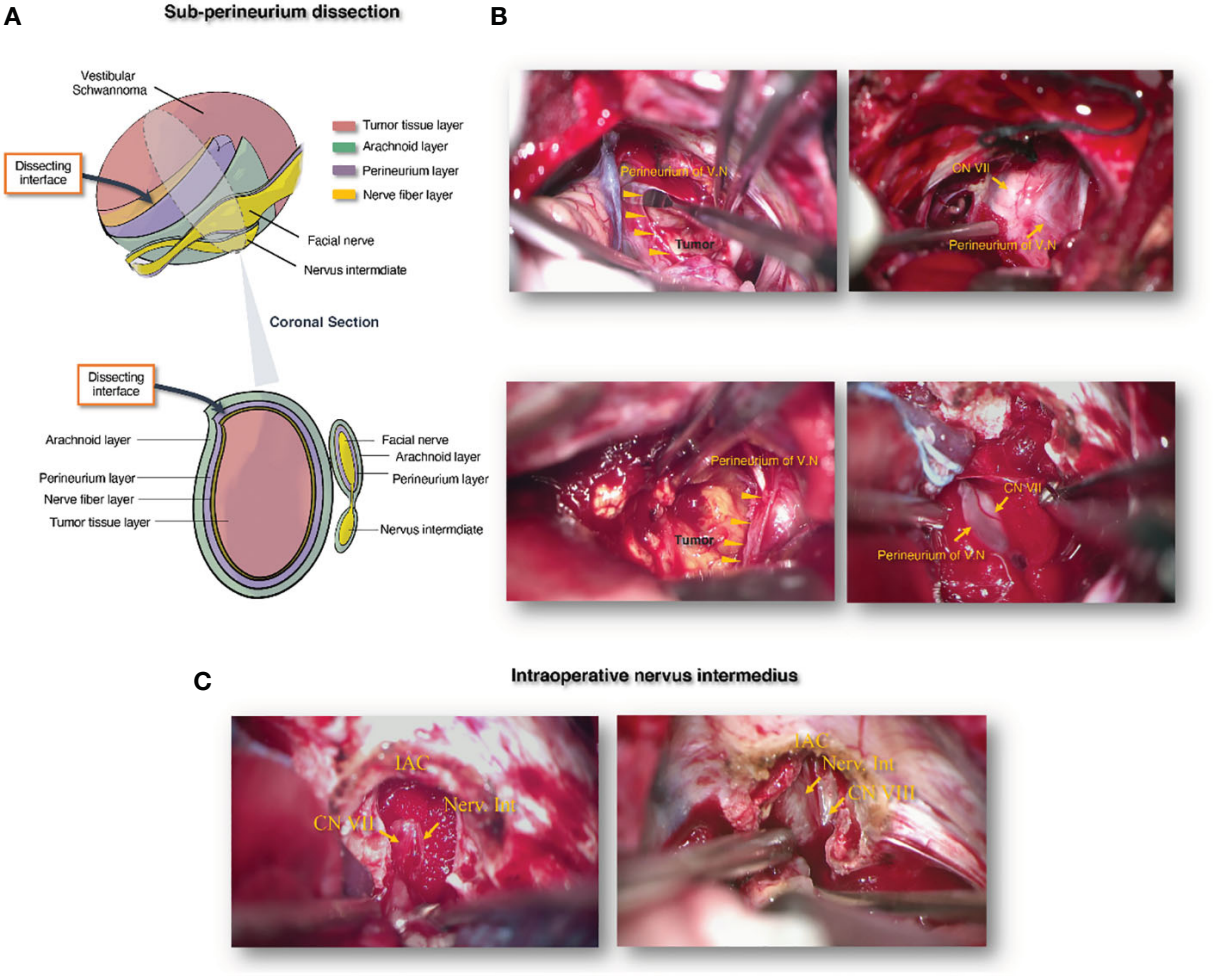
The schematic diagram illustrating the principle of sub-perineurium dissection in vestibular schwannoma microsurgery for neurofunction preservation. **(A)** Schematic diagram of sub-perineurium dissection in vestibular schwannoma microsurgery. **(B)** Intraoperative images of sub-perineurium dissection in vestibular schwannoma microsurgery. **(C)** Intraoperative images of nervus intermedius in vestibular schwannoma microsurgery after IAC management. IAC, internal acoustic canal; CN VII, the 7th cranial nerve; CN VIII: the 8th cranial nerves; Nerv.Int, nervus intermedius.

All 127 patients were surgically treated *via* a retrosigmoid approach, assisted by neurophysiological monitoring of the fifth to 12th cranial nerves. The patients were placed in a lateral supine position. A reverse “L”-shaped incision and musculocutaneous flap were made to expose the asterion and reach inferiorly as far as 2 cm below the inferior nuchal line (INL). A short linear incision was made on the dura to achieve sufficient CSF drainage from the lateral cerebellomedullary cistern, and minimal cerebellar retraction was accomplished. Electrophysiological monitoring was performed during the entire operation of the motor portion of the facial and cochlear nerves. Unless the tumor was classified as Koos Grade I or II, most of the tumors were resected following the rules of vicissitudinary debulking and dissection. Even and adequate debulking (**Figure 1A**) was performed to achieve the result of only a thin layer of tumor tissue being attached to the surrounding neural and vascular structures, which would induce minimal damage to these vital structures. Furthermore, we insisted on obtaining the dissecting plane below the perineurium of the tumor to avoid penetrating the arachnoid layer (**Figures 2A, B**), which may cause injury to the nerves, particularly the NI, because this nerve generally lacks the perineurium itself (**Figure 2A**). After the gross tumor body was removed, the remaining mixed layers of perineurium, arachnoid, and suspicious tumor tissue were eliminated. The integrity and continuity of the facial nerve were further guaranteed by bidirectional dissection (**Figure 1B**): we initially dissected from the brainstem to the IAC, and the posterior wall of the IAC was drilled to open it, following the potential trajectory of CN VII–VIII, to dissect and remove the tumor from the IAC to the brainstem. Finally, the dissection would converge at the prorus acusticus, where the tumor always tightly adheres to the surrounding structure. Following these principles, the NI was not usually visible during the operation because it is thin and located in the extra-perineural region of the vestibular nerve. The integrity and function of the motor portion of the facial nerve were routinely verified by electrophysiological stimulation before removing the retractor, and the cerebellum was re-expanded. The drilled region of the IAC was fixed with a dural substitute and gel foam.

### Surgical outcome

Overall, gross tumor removal was achieved in 126 patients (99.21% of the total). To preserve the anatomical integrity of the facial nerve, subtotal removal was performed in one patient (0.79%) due to the tight adhesion of the tumor to the nerves and the difficulty of separating the mixed membrane structure left on the facial or cochlear nerves. Anatomical continuity of the facial nerve was obtained in 126 patients, except for one case with a thin and widened facial nerve covering the dorsal surface of the tumor.

Preoperatively, 23 of our patients exhibited facial nerve palsy, 21 had HB grade II and two had HB grade III facial palsy. Two months after surgery, 97 (76.38%) patients had normal function of the motor portion of the facial nerve; 25 (19.69%) had HB Grade II, five had HB Grade III (3.94%), and none (0%) had grade IV facial palsy. The incidence of preoperative facial nerve motor injury was almost 20%, which is higher than typically reported; we considered that this was probably because the facial nerve motor had already been affected by tumor compression before surgery, which had a higher Koos grading rate than other reports. The postoperative manifestations of the patients are presented in **Table 2**. In total, 25 patients exhibited only a single symptom: 10 lacrimal disturbances, six dry eyes, five taste disturbances, three xerostomia, and one hypersalivation. Twenty-four patients showed at least two manifestations of NI injury after surgery.

**Table 2:** Postoperative manifestations of NI injury in VS patients.

NI injury manifestation	Lacrimal disturbances	Dry eyes	Taste disturbances	Xerostomia	Hypersalivation	Nasal hypersecretion
Multiple(≥2)	24					
Single (1)	10	6	5	3	1	0

NI, nervus intermedius.

In our case series, the incidence of preoperative NI disturbances was low: dry eyes (0.79%), lacrimal disturbances (0.79%), taste disturbances (3.15%), xerostomia (2.36%), nasal hypersecretion (0%), and hypersalivation (0.79%). Among our patients, the most common postoperative NI dysfunctions were dry eyes (12.60%), lacrimal disturbances (17.32%), taste disturbances (10.24%), xerostomia (7.87%), nasal hypersecretion (3.94%), and hypersalivation (6.30%). The incidence of each symptom of NI dysfunction increased compared to that in the preoperative data. Newly developed NI dysfunction due to surgical management included dry eyes (11.81%), lacrimal disturbances (16.54%), taste disturbances (7.08%), xerostomia (5.51%), nasal hypersecretion (3.94%), and hypersalivation (5.51%) (**Table 3**).

**Table 3:** Summary of the symptoms of preoperative, postoperative, and newly gained NI dysfunction.

Symptoms	Preoperative	Postoperative	Newly gained
lacrimal disturbances	1(0.79%)	22(17.32%)	21(16.54%)
Dry eyes	1(0.79%)	16(12.60%)	15(11.81)
Taste disturbances	4(3.15%)	13(10.24%)	9(7.09%)
Xerostomia	3(2.36%)	10(7.87%)	7(5.51%)
Hypersalivation	1(0.79%)	8(6.30%)	7(5.51)
Nasal hypersecretion	0(0%)	5(3.94%)	5(3.94%)

NI, nervus intermedius.

### Prognostic factors for postoperative NI injury

Baseline characteristics of the patients were obtained from their medical records. Follow-up information was obtained through an online questionnaire and telephonic interviews. Parameters such as sex, age, tumor location, Koos grading, IAC invasion (TFIAC classification), brainstem adhesion, tumor characteristics (cystic or solid), tumor necrosis, preoperative HB facial nerve grading, and extension of tumor resection were collected (**Table 1**). We explored the risk of these parameters for postoperative NI injury using univariate and multivariate analyses. As shown in **Table 4**, the analyses revealed that cystic/solid tumors were strongly associated with NI function after surgical management in both the univariate and multivariate analyses (p <0.01). The Koos grading scale was associated with the prognosis of postoperative NI function in the univariate analysis (p <0.01) but not in the multivariate analysis. However, the other correlation coefficients were not statistically significant in predicting the risk of postoperative NI injury.

**Table 4:** Univariate analysis and multivariate analysis of risk factors for NI injury in VS patients.

Variable	Analysis						
	Univariate analysis			Multivariate analysis			
	F	95%CI	*p*	B	t	95%CI	*p*
Sex	0.329	0.4555-0.6311	0.567	-0.019	-0.256	-0.161-0.124	0.798
Age(years)	2.709	0.4555-0.6311	0.102	-0.021	-0.288	-0.173-0.129	0.774
Tumor location (center/right)	0.004	0.4555-0.6311	0.952	0.022	0.285	-0.129-0.173	0.776
Koos grading scale	7.890	0.4555-0.6311	<0.01	0.244	2.812	0.046-0.266	0.006
Brainstem adhesion	6.154	0.4555-0.6311	0.014	0.013	0.146	-0.209-0.242	0.884
Solid or Cystic tumor	83.591	0.4555-0.6311	<0.01	0.566	7.427	0.443-0.765	<0.01
Tumor necrosis	0.159	0.4555-0.6311	0.691	0.096	1.226	-0.059-0.250	0.223
TFIAC Classification	0.205	0.4555-0.6311	0.893	-0.023	-0.327	-0.076-0.054	0.744
Preoperative HB grading	2.592	0.4555-0.6311	0.079	0.210	0.134	-0.148-0.169	0.893

NI, nervus intermedius.

HB, House-Brackmann.

### Correlation between NI dysfunction and postoperative grade of facial nerve palsy

Disturbances in lacrimal function, taste sensation, as well as xerostomia, hypersalivation, and nasal hypersecretion did not show significant correlations with the postoperative HB grade. These disturbances could occur in patients with normal postoperative functions of the motor portion of the facial nerve. Dry eye incidence between the healthy side and the side of surgery showed a significant correlation with the grade of facial nerve palsy (coefficient = 0.244, p <0.006) (**Table 5**).

**Table 5:** Pearson correlation coefficient of postoperative HB grade and symptoms of NI injury.

Symptoms	PCCs	P
lacrimal disturbances	0.090	0.313
Dry eyes	0.244	0.006*
Taste disturbances	0.141	0.115
Xerostomia	0.147	0.099
Hypersalivation	-0.087	0.329
Nasal hypersecretion	0.056	0.529

PCCs, Pearson correlation coefficient.

## Discussion

### Vestibular schwannoma and NI injury

VSs are common benign intracranial tumors. Many neurosurgical centers have reported an increase in the incidence of VS and the shifting demographic characteristics of affected patients, which is likely due to the rapid development of imaging technology, especially the increasingly widespread access to magnetic resonance imaging (MRI), as opposed to a true biological shift (12–14). Meticulous and sufficient management of VS can provide patients with the most beneficial treatment options. With the accumulation of surgical experience and technological assistance, anatomical preservation of the facial nerve has become the norm today and has been achieved in 93%–98% of cases (5, 15). During outpatient follow-up, even though HB Grades 1–2 were achieved in 91.8% of our VS series, some patients complained about the symptoms of dry eyes, crocodile tears, and dysgeusia, which affected their daily lives due to disturbance to the non-motor portion of the facial nerve. Therefore, attention to NI during tumor resection is important.

The NI, also known as the “nerve of Wrisberg,” was first described by Heinrich August Wrisberg and named the “portio media inter comunicantem faciei et nervum auditorium” (16, 17). Symptoms related to NI dysfunction have been reported soon after microsurgical management. Watanabe et al. found that the number of patients with NI disturbances increased slightly after surgery compared to before surgery (from 28.7% to 34.3%), but the increase was not statistically significant (18). The correlation between the grade of facial nerve palsy and disturbances in NI function showed a discrepancy in the literature. Watanabe et al. and Irving et al. did not find a correlation between the two phenomena, but Stripf et al. reported a positive correlation (9). A prospective study by Samii et al. reported that NI could be affected after VS surgery and was often correlated with dysfunction of the motor portion of the facial nerve. NI disability should be evaluated and reported separately while analyzing facial nerve function after VS surgery (19). Noonan et al. reported the clinical importance of monitoring NI symptoms because a high percentage of all patients undergoing intervention are symptomatic during management. Their study showed that patients with facial nerve palsy were at a higher risk of developing NI sequelae (20). Data from the study by Stripf et al. demonstrated the clinical importance of NI defects associated with CPA tumor removal. More than 50% of patients in this study reported postoperative NI disturbances. These sequelae may affect both the short- and long-term postoperative quality of life to the same extent as deafness or transient facial paresis. The risk of injury appears to be higher after the middle fossa approach (9).

Regarding radiotherapy, Park et al. and Stripf et al. demonstrated that patients undergoing radiotherapy for VS experience various disturbances of the NI, which is useful information about the likelihood of certain post-surgical symptoms for VS (21).

To the best of our knowledge, no detailed analysis of the risk factors affecting the outcome of NI preservation before surgery has been reported. We found that cystic or solid tumors were strongly associated with NI function after surgical management in both univariate and multivariate analyses (p <0.01), which corresponds to our clinical experience. Nerve protection is much more difficult for cystic VSs because once the fluid is released from the cyst, the mass effect of the original tumor disappears and only an extremely thin tumor capsule adheres to the nerves (B = 0.566). Hence, the trajectories of the facial and cochlear nerves may change dramatically and become undetectable. Meanwhile, the dissection interface becomes much more difficult to identify. The Koos grading scale was associated with the prognosis of postoperative NI functions in the univariate analysis, but not in the multivariate analysis (p <0.01). Koos grading has been widely utilized in clinical assessments and has been verified as a risk factor for NI injury after surgery. The VSs of Koos grade IV are always difficult to handle; in particular, the nerve origin from the brainstem is difficult to expose because of the adhesion between the tumor and brainstem (B = 0.244).

The NI is much thinner than the motor portion of the facial nerve, and it has different connections with adjacent nerves in the CPA. It also has no perineurium and is generally covered with just a thin layer of arachnoidea when coursing in the CPA cistern, making it vulnerable to mechanical damage. In addition, the various orientations, tortuous trajectories, and flexible anastomoses of the NI make it more difficult to preserve than other nerves in the vicinity of the tumor (8, 22). For example, NIs attached to the superior or inferior vestibular nerves are easily injured during surgery and cannot be preserved in some cases. Therefore, pertinent strategies and crafted techniques can ameliorate the risk of NI damage and further optimize the quality of the patient’s life.

According to the Koos grading scale for VS, the 127 cases in this study contained more than 98.43% of grade II to grade IV VSs. Of note, the two patients with HB grade III suffered strokes due to tumor prior to surgery, so their facial nerves were more easily disturbed. Thus, our cases exhibited a higher incidence of preoperative facial nerve motor injury than is typically reported. Given the higher motor dysfunction preoperatively, the incidence of preoperative NI dysfunction was relatively low. The NI is multifunctional and carries the sensory and parasympathetic functions of the facial nerve. In our opinion, the various orientations, tortuous trajectories, and flexible anastomoses of the NI make it less susceptible to dysfunction caused by the tumor preoperatively. However, studies with larger case series and more objective examinations are required.

### Principle of adequate and even debulking

The tumor should be debulked piecemeal, evenly, and three-dimensionally, which can effectively reduce its extrusion effect on the surrounding tissues. After the tumor volume is reduced, the interface between the tumor and the neurovascular structure can be identified, and safe separation is feasible. In the process of continuous debulking and dissection, evoked potential nerve monitoring is performed to avoid accidental injury when distinguishing tumor tissues from nerves. When the tumor is adequately decompressed, it is usually possible to identify the origin of the facial nerve from the brainstem in the ventral portion of the tumor, where the facial nerve is usually displaced. Taking the brainstem end of the facial nerve, the cerebellar flocculus, the choroid plexus of Luschka’s foramen, and the IAC as anatomical landmarks, alternate debulking and dissection along the trajectory can avoid uneven mechanical forces on the delicate nerve fibers.

### Principle of sub-perineurium dissection

Based on even and adequate debulking, only thin mixed layers composed mainly of different types of tissues are left between the tumor and CN VII–VIII, which helps to expose the safe interface with minimal stretching. There are sometimes thin arachnoid layers between the cisternal CN VII–VIII and the tumor originating from the vestibular nerve in the cerebellopontine cistern. Notably, this interface may disappear owing to the special pathological behavior of some individual tumors; thus, it is easy to damage the nerves following subarachnoid dissection. However, histopathological studies have shown that VS has a “capsule” composed of the vestibular perineurium and nerve fibers (23). We believe that dissection should be strictly restricted under the perineurium (**Figures 2A, B**), especially when the tumor tightly adheres to the nerves. Safe dissection between the perineurium and residual vestibular nerve fibers can avoid damaging the facial and cochlear nerves. In the end, the extremely thin membrane left on CN VII–VIII may contain residual tumor tissue after the main tumor body has been removed, so we usually cautiously peel off this thin membrane in case of tumor recurrence. Nevertheless, for functional preservation, subtotal resection is inevitable when the thin membrane adheres too tightly to the nerves. Naturally, these special cases were closely observed.

### Principle of bidirectional dissection

Except for the cases of Koos grade I, we performed bidirectional dissection for all the other VSs protruding into the CPA cistern (**Figure 1B**). First, we identified the origin of CN VII–VIII at the brainstem, followed by careful separation between the thin tumor tissue and nerves without piercing the perineurium, along the “corridor” from the brainstem to the IAC, guided by the trajectory of the facial nerve. The region of the porous acoustics is typically associated with the highest risk of facial nerve injury during CPA surgery due to the extremely tight adhesion between the nerves and tumor tissue (5). Although the shape of the NI and the motor portion of the facial nerve are visible, we always suspend the one-way dissection and drill the posterior wall of the IAC to make it appropriately open according to the preoperative imaging. The residual tumor extending into the IAC is gently pulled out with a nerve stripper to expose the anatomical structure of CN VII–VIII, which is usually located between the motor portion of the facial and cochlear nerves (**Figure 2C**). Finally, the dissection converges at the porous acoustics from both directions; therefore, the last adhesion point of the tumor with a normal structure can be amputated safely. In our opinion, bidirectional dissection with proper IAC opening minimizes the risk of NI injury; thus, we did not identify a significant correlation with IAC invasion (TFIAC classification).

### The idea of *in situ* dissection between the nerve and tumor

Under the principles of “adequate and even debulking” and “sub-perineurium and bidirectional dissection,” we propose the concept of *in situ* dissection between nerves and tumors. In brief, after intratumoral debulking, only thin layers of mixed tissue composed of tumor cells, a small amount of vestibular nerve fibers, and the vestibular perineurium attach to the neurovascular structures. At this time, the facial and cochlear nerves should be maintained *in situ* in their original anatomical positions and attempts to stretch nerve structures away from the tumor should always be avoided during the entire operation. In contrast, we peeled the thin layer of tumor tissue from the facial and cochlear nerves under the vestibular perineurium. This was done with as minimal displacement of the nerves as possible. Therefore, no force is exerted on the thin and delicate nerves. During the operation, after the last adhesion between the tumor and facial nerve is peeled off, satisfactory nerve function preservation is indicated when no serious spontaneous electromyographic response occurs and complete electromyographic waves can be induced. However, electrophysiological monitoring of the NI is still under trial and has not been widely utilized in clinical practice (24).

## Conclusion

Little attention has been given to NI dysfunction following VS surgery. However, it affects patients’ quality of life. Although the motor function of the facial nerve is well preserved, NI disturbances are still common after VS surgery. The continuity of the facial nerve is the premise of its motor function, and we propose maintaining the integrity of the facial nerve to preserve NI function. Performing bidirectional and sub-perineurium dissection based on even and adequate debulking is the key principle for NI preservation in VS surgery. Notably, a higher Koos grading and cystic characteristics of VS are strongly associated with postoperative NI injury. These two parameters can be used to guide the development of a surgical strategy and predict the prognosis for NI function preservation.

## Data availability statement

The original contributions presented in the study are included in the article/supplementary material. Further inquiries can be directed to the corresponding authors.

## Ethics statement

All surgical procedures were reviewed and approved by the ethics committee of Xiangya Hospital and the patients’ family members, who provided written informed consent to participate in this study and are able to share their perspectives.

## Author contributions

CQ, QL and WL performed the surgical procedures. SZ, YuL, YP, YaL, CW, KX, JS and XW performed data collection and analysis. YuL, HP, CQ and QL wrote the manuscript. QL and CQ supervised the entire work. All the authors provided final approval for the version to be published.

## References

[1] Carlson ML Link MJ. Vestibular schwannomas. N Engl J Med (2021) 384(14):1335–48. doi: 10.1056/NEJMra2020394 33826821

[2] Ansari SF Terry C Cohen-Gadol AA. Surgery for vestibular schwannomas: a systematic review of complications by approach. Neurosurg Focus (2012) 33(3):E14. doi: 10.3171/2012.6.FOCUS12163 22937848

[3] Gauden A Weir P Hawthorne G Kaye A. Systematic review of quality of life in the management of vestibular schwannoma. J Clin Neurosci (2011) 18(12):1573–84. doi: 10.1016/j.jocn.2011.05.009 22014598

[4] Samii M Matthies C. Management of 1000 vestibular schwannomas (acoustic neuromas): surgical management and results with an emphasis on complications and how to avoid them. Neurosurgery (1997) 40(1):11–21; discussion -3. doi: 10.1227/00006123-199701000-00002 8971819

[5] Samii M Gerganov V Samii A. Improved preservation of hearing and facial nerve function in vestibular schwannoma surgery *via* the retrosigmoid approach in a series of 200 patients. J Neurosurg (2006) 105(4):527–35. doi: 10.3171/jns.2006.105.4.527 17044553

[6] Samii M Matthies C Tatagiba M. Management of vestibular schwannomas (acoustic neuromas): auditory and facial nerve function after resection of 120 vestibular schwannomas in patients with neurofibromatosis 2. Neurosurgery (1997) 40(4):696–705; discussion -6. doi: 10.1097/00006123-199704000-00007 9092842

[7] Alfieri A Fleischhammer J Prell J. The functions of the nervus intermedius. AJNR Am J Neuroradiol (2011) 32(7):E144. doi: 10.3174/ajnr.A2624 21724571 PMC7966028

[8] Alfieri A Fleischhammer J Peschke E Strauss C. The nervus intermedius as a variable landmark and critical structure in cerebellopontine angle surgery: an anatomical study and classification. Acta Neurochir (Wien) (2012) 154(7):1263–8. doi: 10.1007/s00701-012-1359-4 22555552

[9] Stripf T Braun K Gouveris H Stripf EA Mann WJ Amedee RG. Influence of different approaches to the cerebellopontine angle on the function of the intermediate nerve. J Neurosurg (2007) 107(5):927–31. doi: 10.3171/JNS-07/11/0927 17977262

[10] Kang TS Vrabec JT Giddings N Terris DJ. Facial nerve grading systems (1985-2002): beyond the house-brackmann scale. Otol Neurotol (2002) 23(5):767–71. doi: 10.1097/00129492-200209000-00026 12218632

[11] Zhou W Wang Y Ma S Yuan L Wang X Peng J. A novel imaging grading biomarker for predicting hearing loss in acoustic neuromas. Clin Neuroradiol (2020) 31:599–610. doi: 10.1007/s00062-020-00938-7 32720068

[12] Marinelli JP Lohse CM Grossardt BR Lane JI Carlson ML. Rising incidence of sporadic vestibular schwannoma: True biological shift versus simply greater detection. Otol Neurotol (2020) 41(6):813–47. doi: 10.1097/MAO.0000000000002626 PMC7311288 32150020

[13] Marinelli JP Grossardt BR Lohse CM Carlson ML. Prevalence of sporadic vestibular schwannoma: Reconciling temporal bone, radiologic, and population-based studies. Otol Neurotol (2019) 40(3):384–90. doi: 10.1097/MAO.0000000000002110 PMC7245016 30688755

[14] Reznitsky M Petersen M West N Stangerup SE Caye-Thomasen P. Epidemiology of vestibular schwannomas - prospective 40-year data from an unselected national cohort. Clin Epidemiol (2019) 11:981–6. doi: 10.2147/CLEP.S218670 PMC6850685 31807080

[15] Gormley WB Sekhar LN Wright DC Kamerer D Schessel D. Acoustic neuromas: results of current surgical management. Neurosurgery (1997) 41(1):50–8; discussion 8-60. doi: 10.1097/00006123-199707000-00012 9218295

[16] Alfieri A Strauss C Prell J Peschke E. History of the nervus intermedius of wrisberg. Ann Anat (2010) 192(3):139–44. doi: 10.1016/j.aanat.2010.02.004 20427169

[17] Tubbs RS Steck DT Mortazavi MM Cohen-Gadol AA. The nervus intermedius: a review of its anatomy, function, pathology, and role in neurosurgery. World Neurosurg (2013) 79(5-6):763–7. doi: 10.1016/j.wneu.2012.03.023 22484073

[18] Watanabe K Saito N Taniguchi M Kirino T Sasaki T. Analysis of taste disturbance before and after surgery in patients with vestibular schwannoma. J 5Neurosurg (2003) 99(6):999–1003. doi: 10.3171/jns.2003.99.6.0999 14705727

[19] Metwali H Kniese K Kardavani B Gerganov V Samii M. Nervus intermedius dysfunctions after vestibular schwannoma surgery: A prospective clinical study. J Neurosurg (2018) 131(2):555–60. doi: 10.3171/2018.4.JNS1818 30192193

[20] Noonan KY Rang C Callahan K Simmons NE Erkmen K Saunders JE. Nervus intermedius symptoms following surgical or radiation therapy for vestibular schwannoma. Otolaryngol Head Neck Surg (2016) 155(4):657–62. doi: 10.1177/0194599816655144 27301896

[21] Park SH Lee KY Hwang SK. Nervus intermedius dysfunction following gamma knife surgery for vestibular schwannoma. J Neurosurg (2013) 118(3):566–70. doi: 10.3171/2012.10.JNS12747 23101447

[22] Irving RM Viani L Hardy DG Baguley DM Moffat DA. Nervus intermedius function after vestibular schwannoma removal: clinical features and pathophysiological mechanisms. Laryngoscope (1995) 105(8 Pt 1):809–13. doi: 10.1288/00005537-199508000-00007 7630291

[23] Sasaki T Shono T Hashiguchi K Yoshida F Suzuki SO. Histological considerations of the cleavage plane for preservation of facial and cochlear nerve functions in vestibular schwannoma surgery. J Neurosurg (2009) 110(4):648–55. doi: 10.3171/2008.4.17514 18928359

[24] Ashram YA Jackler RK Pitts LH Yingling CD. Intraoperative electrophysiologic identification of the nervus intermedius. Otol Neurotol (2005) 26(2):274–9. doi: 10.1097/00129492-200503000-00026 15793419

